# Daunorubicin reduces MBNL1 sequestration caused by CUG-repeat expansion and rescues cardiac dysfunctions in a *Drosophila* model of myotonic dystrophy

**DOI:** 10.1242/dmm.032557

**Published:** 2018-04-23

**Authors:** Mouli Chakraborty, Chantal Sellier, Michel Ney, Villa Pascal, Nicolas Charlet-Berguerand, Ruben Artero, Beatriz Llamusi

**Affiliations:** 1 Translational Genomics Group, Incliva Health Research Institute, Valencia, Spain; 2 Department of Genetics and Interdisciplinary Research Structure for Biotechnology and Biomedicine (ERI BIOTECMED), University of Valencia, Valencia 46100, Spain; 3 CIPF-INCLIVA Joint Unit, Valencia 46100, Spain; 4 Translational Medicine and Neurogenetics, Institut de Génétique et de Biologie Moléculaire et Cellulaire (IGBMC), INSERM U964, CNRS UMR7104, University of Strasbourg, 1 Rue Laurent Fries, 67400 Illkirch-Graffenstaden, France; 5 PCBIS Plate-forme de Chimie Biologique Intégrative de Strasbourg CNRS UMS 3286, Labex Medalis, ESBS, Université de Strasbourg, 300 Boulevard Sébastien Brant, 67412 Illkirch, France

**Keywords:** Daunorubicin, *Drosophila*, Muscleblind, Myotonic dystrophy, Trinucleotide repeat disorder

## Abstract

Myotonic dystrophy (DM) is a dominantly inherited neuromuscular disorder caused by expression of mutant myotonin-protein kinase (*DMPK*) transcripts containing expanded CUG repeats. Pathogenic *DMPK* RNA sequesters the muscleblind-like (MBNL) proteins, causing alterations in metabolism of various RNAs. Cardiac dysfunction represents the second most common cause of death in DM type 1 (DM1) patients. However, the contribution of MBNL sequestration in DM1 cardiac dysfunction is unclear. We overexpressed *Muscleblind* (*Mbl*), the *Drosophila* *MBNL* orthologue, in cardiomyocytes of DM1 model flies and observed a rescue of heart dysfunctions, which are characteristic of these model flies and resemble cardiac defects observed in patients. We also identified a drug – daunorubicin hydrochloride – that directly binds to CUG repeats and alleviates *Mbl* sequestration in *Drosophila* DM1 cardiomyocytes, resulting in mis-splicing rescue and cardiac function recovery. These results demonstrate the relevance of Mbl sequestration caused by expanded-CUG-repeat RNA in cardiac dysfunctions in DM1, and highlight the potential of strategies aimed at inhibiting this protein-RNA interaction to recover normal cardiac function.

## Abstract (precis)

**Summary:** MBNL protein sequestration by expanded CUG RNA contributes towards cardiac dysfunction in a myotonic dystrophy *Drosophila* model. Here, the authors identify the anticancer drug daunorubicin as a candidate therapeutic for the disease.

## INTRODUCTION

Myotonic dystrophy type 1 [DM1; Online Mendelian Inheritance of Man (OMIM) 160900] is the most common muscular dystrophy in adults (Harper, 2001; Smith and Gutmann, 2016). Presently, DM1 has no effective treatment, but several therapeutic options are being explored (Rzuczek et al., 2017; Gao et al., 2016; Bisset et al., 2015; Konieczny et al., 2017). Although DM1 mainly affects skeletal muscle, cardiac involvement occurs in 80% of DM1 patients and represents the second most common cause of death after respiratory failure (Vinereanu et al., 2004). Three interrelated cardiac phenotypes are observed in individuals with DM1. The first are conduction defects, which can progress to complete heart blockage (Nguyen et al., 1988). The second is the development of potentially fatal ventricular and/or atrial arrhythmias (Nigro et al., 2012; Benhayon et al., 2015). The third type of cardiac dysfunction observed in DM1, although rarer, is mechanical diastolic and/or systolic dysfunction that can progress to combined systolic and diastolic heart failure (Phillips and Harper, 1997; Mathieu et al., 1999; Lazarus et al., 2002; Groh et al., 2008). The genetic cause of DM1 is an expansion of CTG repeats in the 3′UTR of the *DMPK* gene (Brook et al., 1992; Fu et al., 1992; Mahadevan et al., 1992). This microsatellite expansion is transcribed into mutant *DMPK* mRNA that contains hundreds to thousands of CUG repeats that are toxic through dysfunction of at least two RNA-binding proteins. The muscleblind-like (MBNL) family of proteins, comprising MBNL1, MBNL2 and MBNL3, normally bind to YGC (Y stands for pyrimidine) RNA motifs and are diverted away from their normal RNA targets by the expanded CUG RNA repeats in *DMPK* transcripts (Miller et al., 2000; Mankodi et al., 2001; Fardaei et al., 2002; Ho et al., 2004; Lin et al., 2006; Wang et al., 2015). Furthermore, expanded CUG repeats also induce hyperphosphorylation and increase stabilization of CUG-binding protein 1 (CELF1; also named CUGBP1) (Kuyumcu-Martinez et al., 2007). As a result of disrupting the function of these proteins, various mis-regulations in RNA metabolism have been described in individuals with DM1, some of which are associated with specific symptoms of the disease (Mankodi et al., 2002; Savkur et al., 2001; Tang et al., 2012; Fugier et al., 2011; Freyermuth et al., 2016).

Reduced MBNL function appears to be a key pathogenic event underlying the skeletal muscle alterations observed in DM1 (Kanadia et al., 2003; Nakamori et al., 2013; Wagner et al., 2016). Indeed, overexpression of MBNL1 rescues DM1-like skeletal muscle alterations in a mouse model of DM1 (Kanadia et al., 2006). Furthermore, compounds or strategies aimed at reducing MBNL1 binding to expanded CUG repeats alleviate skeletal muscle dysfunctions in cell and animal models of DM1 (Warf et al., 2009; García-López et al., 2011; Childs-Disney et al., 2013; Cerro-Herreros et al., 2016). In contrast, the molecular causes of cardiac dysfunction in DM1 and the involvement of MBNL proteins in these defects are not yet fully understood. Notably, compound loss of Mbnl1 and Mbnl2 in mice generated cardinal features of DM1, including heart conduction dysfunction (Lee et al., 2013). However, elevation of CUGBP1 is an early event in the heart of a mouse model of DM1 (Wang et al., 2007), and heart-specific overexpression of CUGBP1 in mice induces functional and molecular alterations (Koshelev et al., 2010). Furthermore, inhibition of CUGBP1 hyperphosphorylation ameliorates the cardiac phenotype in a mouse model of DM1 (Wang et al., 2009). Importantly, both MBNL and CUGBP1 are master switches for normal heart development as they regulate a large subset of alternative splicing transitions that occur post-natally (Wang et al., 2015; Kalsotra et al., 2008). These data suggest that the pathogenic events underlying the cardiac dysfunctions in DM1 remain to be fully defined.

To study the importance of MBNL proteins in the heart dysfunction induced by expanded CUG repeats in *DMPK*, we used a *Drosophila* model expressing pure expanded CUG repeats (250 CUG repeats) in cardiomyocytes (Chakraborty et al., 2015). Importantly, cardiac dysfunction described in these flies is completely reversed by the sole overexpression of Mbl, the *Drosophila* orthologue of MBNL proteins. To further support the relevance of this protein in DM1 cardiac dysfunction, we searched *in vitro* for compounds that inhibit the interaction between MBNL1 and expanded CUG repeats. One of these compounds, daunorubicin hydrochloride, releases endogenous Mbl from CUG RNA foci by directly binding to CUG RNA, resulting in the correction of Mbl-dependent splicing alterations and subsequent recovery of heart dysfunction and survival in DM1 flies. These data highlight not only the relevance of MBNL depletion related to heart dysfunction in DM1, but also provide evidence that strategies aimed at releasing MBNL proteins from expanded CUG RNA repeats might also be useful to treat cardiac dysfunction in DM1.

## RESULTS

### Muscleblind is sufficient to rescue the cardiac dysfunctions and reduced survival caused by expanded CUG repeats

**Fig. 1.**
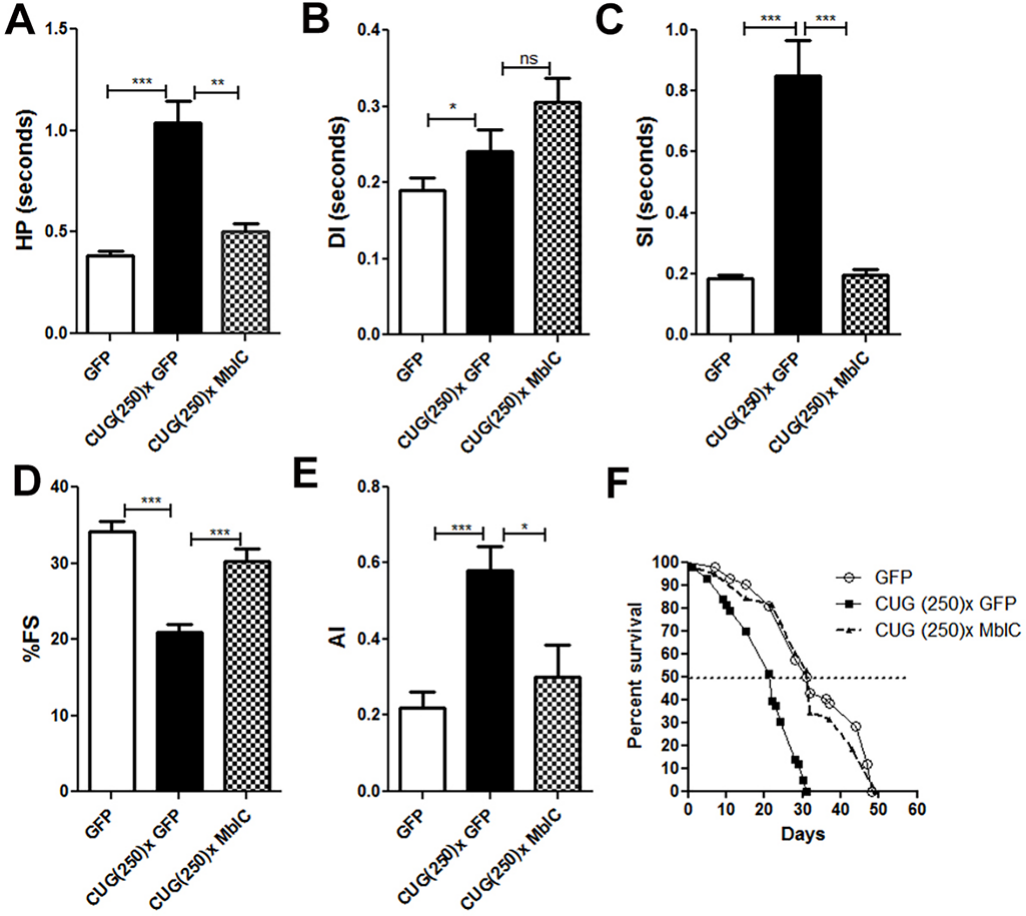
**Cardiac dysfunction and survival are rescued by MblC overexpression in DM1 model flies.** In comparison to control flies expressing GFP, the heart period mean (HP; in A) increased in flies expressing expanded CUG repeats and GFP [CUG(250)× GFP] together. Increased HP is caused by extended diastolic and systolic intervals (DI and SI; in B and C, respectively). In contrast, simultaneous expression of CUG repeats and MblC [CUG(250)× MblC] achieved a reduction of HP length by a large decrease of SI. Heart contractility, measured as percentage of fractional shortening (%FS; in D), and arrhythmicity, measured as arrhythmia index (AI; in E), which were altered in DM1 flies, were also rescued by MblC overexpression (*n*=18 to 29). Median survival and lifespan were also rescued by MblC overexpression (F; *n*≈50). ***P<0.001, **P<0.01, *P<0.05, ns, not significant (Student's *t*-test).

To understand the role of Mbl in cardiac dysfunction induced by expanded CUG repeat expression, we generated recombinant flies that simultaneously expressed 250 pure CUG repeats and either Mbl isoform C (MblC; the best evolutionary conserved Mbl isoform in *Drosophila*) or green fluorescent protein (GFP), as control. Cardiac parameters were analyzed using semi-automated optical heartbeat analysis (SOHA) software on 7-day-old female flies as described previously (Chakraborty et al., 2015; Ocorr et al., 2014; Cerro-Herreros et al., 2017). Heart dysfunctions, including systolic and diastolic dysfunction, arrhythmia and reduced contractility, in DM1 model flies expressing GFP were identical to DM1 flies (Fig. S1) and were similar to the previously reported alterations (Chakraborty et al., 2015), indicating that the expression of the GFP reporter is innocuous in heart. In contrast to GFP, expression of MblC corrects the cardiac parameters of DM1 flies: *Drosophila* expressing 250 pure CUG repeats and MblC were similar to control flies that do not express the repeats. Notably, MblC expression rescued heart period length [HP; defined as the diastolic interval (DI) plus systolic interval (SI)] (Fig. 1A-C), mainly by a 2-fold reduction of the SI (the contraction period). The variability in the heart periodicity, quantified as ‘arrhythmia index’ (AI), and the heart contractility, or percentage of fractional shortening (%FS), were also rescued in DM1 flies by MblC overexpression (Fig. 1D,E). Finally, survival, which was decreased in DM1 model flies, was also corrected by MblC, with median survival increasing from 22 to 32 days, and the lifespan increasing from 31 to 50 days (Fig. 1F). These results support an important role of Mbl loss of function in the heart dysfunctions induced by expression of expanded CUG repeats.

### Identification of compounds reducing MBNL1 binding to CUG repeats

**Fig. 2.**
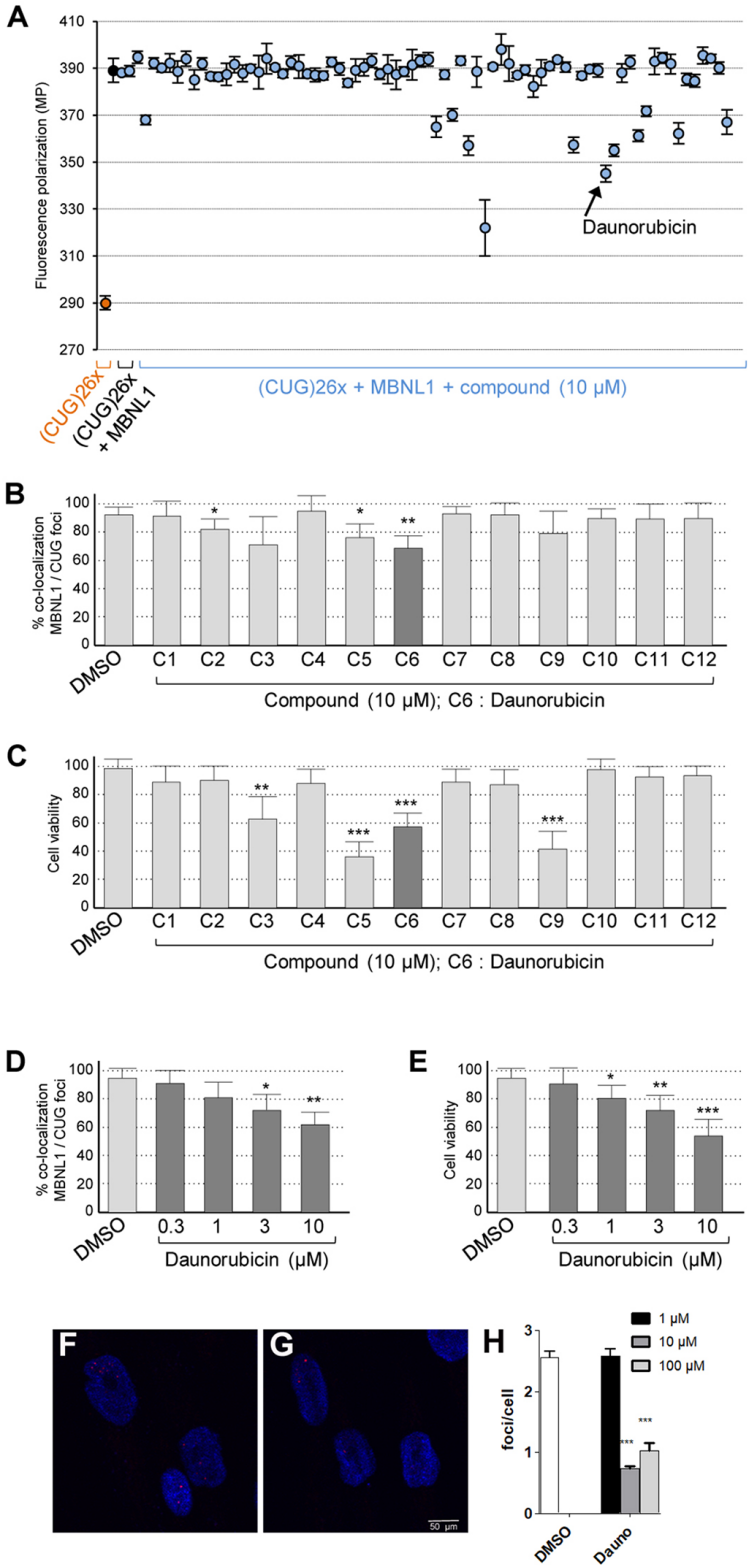
**Validation screening of the 76 compounds tested at a concentration of 10 µM in DMSO to identify molecules reducing binding of MBNL1 to expanded-CUG-repeat RNA.** (A) Fluorescence polarization of TRITC-labelled (CUG)26× RNA alone or in complex with GST-MBNL1-HIS recombinant protein is indicated by orange or blue dots, respectively. *N*=3 independent assays. (B) Percent of CUG RNA foci presenting a colocalization with endogenous MBNL1 in cultures of DM1 myoblasts upon drug treatment at 10 µM in DMSO for 24 h. *N*=3 independent cultures; 30 RNA foci were analyzed in each experiment. (C) Percent of living DM1 myoblasts upon drug treatment at 10 µM in DMSO for 24 h. (D) Percent of CUG RNA foci showing colocalization with endogenous MBNL1 in cultures of DM1 myoblasts upon daunorubicin treatment at 0.3, 1, 3 and 10 µM in DMSO for 24 h. *N*=4 independent cultures; at least 50 cells were analyzed each time. (E) Percent of living DM1 myoblasts upon daunorubicin treatment at 0.3, 1, 3 and 10 µM in DMSO for 24 h. (F-H) Foci detection in DM1 fibroblasts. Representative confocal images of FISH in DM1 fibroblasts treated with DMSO as control (F) or daunorubicin (G) showed reduced number of foci (red in F and G) with daunorubicin treatment. Nuclei were counterstained with DAPI (blue). (H) Quantification of foci confirmed a statistically significant difference at concentrations of daunorubicin higher than 1 µM. For all figure panels, error bars indicate s.e.m. Student's *t*-test: *P<0.05; **P<0.01; ***P<0.001.

To confirm the relevance of MBNL loss of function in cardiac dysfunctions in DM1, we developed a real-time fluorescence polarization/anisotropy assay to identify pharmacological compounds reducing MBNL1 binding to pathogenic CUG repeats. Fluorescence polarization measures the degree of polarization of a fluorophore, reflecting its rotational diffusion, a parameter that is inversely proportional to the molecule volume and thus reflects the molecular mass of the fluorescent-labelled molecule or complex. In short, fluorescence polarization can discriminate between a fluorescent-labelled RNA free in solution compared to the same RNA in complex with an RNA-binding protein. Consequently, a molecule that disrupts the binding between a fluorescent RNA and a protein can be identified by a reduction of the fluorescence polarization. In our assay, we used a chemically synthetized CUG repeat (26 repeats) RNA labelled with a tetramethylrhodamine and incubated with purified recombinant GST-MBNL1-HIS produced from *Escherichia coli*. An optimal shift of fluorescent polarization was observed with 50 nM of fluorescent CUG RNA incubated with 400 nM of recombinant MBNL1 protein. We screened in 96-well plates and at 10 µM final concentration ∼6500 compounds from two libraries, the Strasbourg Academic library, consisting of ∼5300 chemical or natural substances, and the Prestwick library, consisting of 1200 drugs approved by regulatory agencies. From this screen, we identified 76 molecules, of which 12 were verified by fluorescent polarization to inhibit binding of MBNL1 to CUG-repeat RNA *in vitro* (Fig. 2A). Next, we tested whether these compounds can reduce the sequestration of MBNL1 in human cultured cells. The colocalization of endogenous MBNL1 with foci of expanded CUG RNA was assessed by immunofluorescence coupled to RNA fluorescent *in situ* hybridisation (FISH) in primary cultures of myoblasts originated from a muscle biopsy of an individual with DM1. Among the 12 compounds tested, three compounds – C2, C5 and C6 – decreased the sequestration of MBNL1 *in cellulo* (Fig. 2B). However, C5 was toxic and not investigated further (Fig. 2C). Subsequent studies in DM1 primary muscle cell cultures revealed that compound C6, daunorubicin hydrochloride, was achieving the most important reduction of MBNL1 colocalization with CUG RNA foci. Addition of 3-10 µM of daunorubicin reduced the colocalization of MBNL1 with CUG RNA foci by 20-30% in DM1 primary muscle cell cultures, but with some toxicity (Fig. 2D,E). Of note, daunorubicin treatment of control or DM1 primary muscle cell cultures did not modify protein expression of MBNL1, nor the mRNA expression of *DMPK* (Fig. S2A,B). We also quantified foci number in human DM1 fibroblasts (Arandel et al., 2017) at increasing concentrations of daunorubicin, and observed a significant reduction of foci number at concentrations higher than 1 µM (Fig. 2F-H). These data encouraged us to test whether daunorubicin could correct any phenotypic alterations in an animal model of DM1.

### Daunorubicin rescues cardiac dysfunction and fly survival in DM1 model flies

**Fig. 3.**
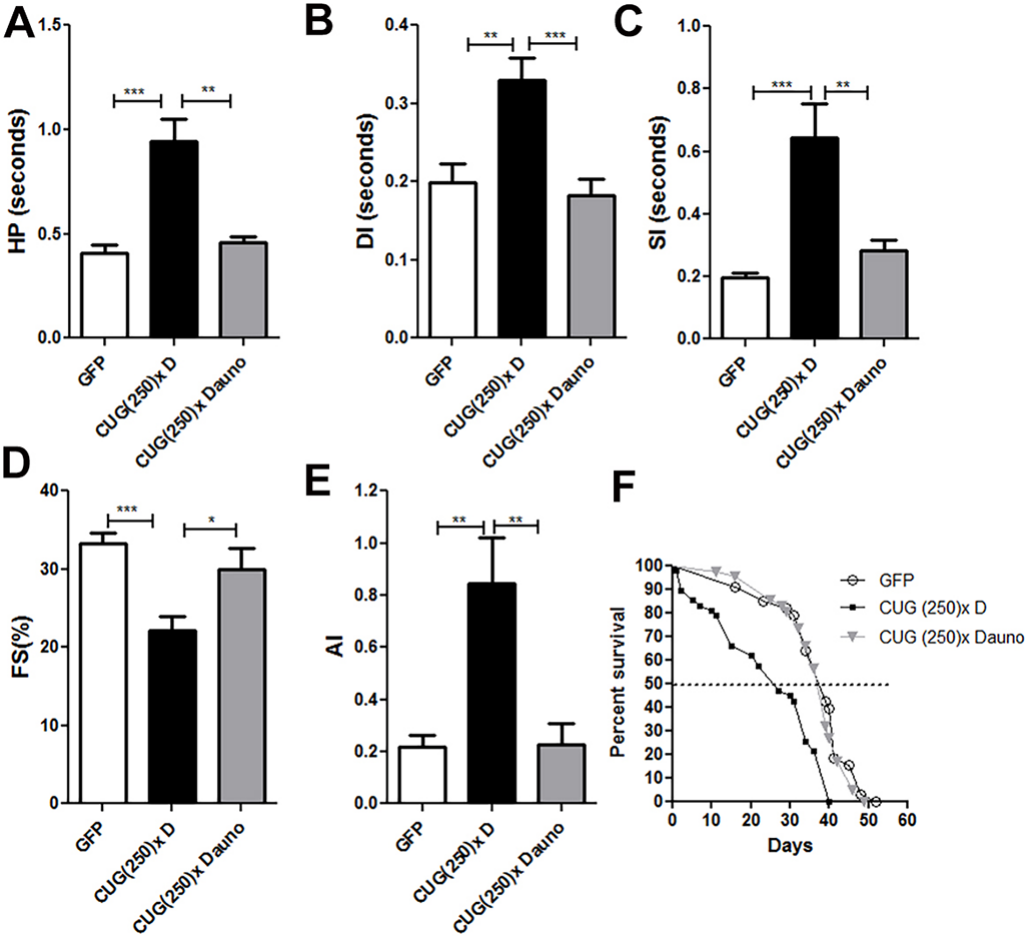
**All DM1 cardiac alterations were rescued by daunorubicin.** (A-E) Alterations in HP, SI, DI, %FS and AI found in model flies fed with DMSO (‘D’) were significantly rescued to nearly normal conditions by treatment with daunorubicin hydrochloride (Dauno) (*n*=14 to 29). (F) Median survival was also rescued by daunorubicin treatment (*n*≈50). ***P<0.001, **P<0.01, *P<0.05 (Student's *t*-test).

Daunorubicin is a double-stranded DNA or RNA intercalant that belongs to the anthracyclines family. It has been used as curative or palliative treatment for several types of cancer for over 30 years (Hande, 1998; Gewirtz, 1999; Ellison et al., 1991; Kоbylinska et al., 2016). To assess the potential of daunorubicin to rescue DM1 cardiac phenotypes, we fed DM1 model flies with the compound diluted in 1% DMSO to a final concentration of 1 μM in nutritive media. We had previously shown that, at the concentration used, DMSO does not alter the cardiac parameters in *Drosophila* (Chakraborty et al., 2015). For cardiac function assessment, 7-day-old flies fed with the compound were dissected to expose the beating heart in artificial aerated haemolymph, and videos were taken and analyzed by SOHA software (Chakraborty et al., 2015; Ocorr et al., 2014; Cerro-Herreros et al., 2017). In all analyses, flies that do not express CUG repeats but expressed GFP were used as controls. As compared to the DMSO-fed model flies, flies fed with daunorubicin showed significant improvement in HP length (Fig. 3A), with a significant 2-fold decrease in both the DI and SI (Fig. 3B,C). Furthermore, daunorubicin corrected AI (Fig. 3E), which decreased by almost 3-fold in treated flies, and rescued heart contractility, assessed by the calculation of %FS (Fig. 3D). Finally, DM1 model flies fed with DMSO had a median survival of only 28 days, while survival of DM1 flies fed with daunorubicin increased up to 40 days, which is close to the 47 days of median survival in control flies with no CTG repeats (Fig. 3F). As a control to discard a general effect of daunorubicin on flies’ survival, we fed flies expressing GFP in cardiac muscle with daunorubicin and analyzed their survival curves. Daunorubicin had no effect on the survival of these control flies (Fig. S3A). In conclusion, daunorubicin improved all the cardiac parameters of DM1 flies, as well as their median survival, but did not affect control flies.

### Daunorubicin redistributed Mbl from foci in *Drosophila* cardiomyocytes and rescued Mbl-dependent splicing defects

**Fig. 4.**
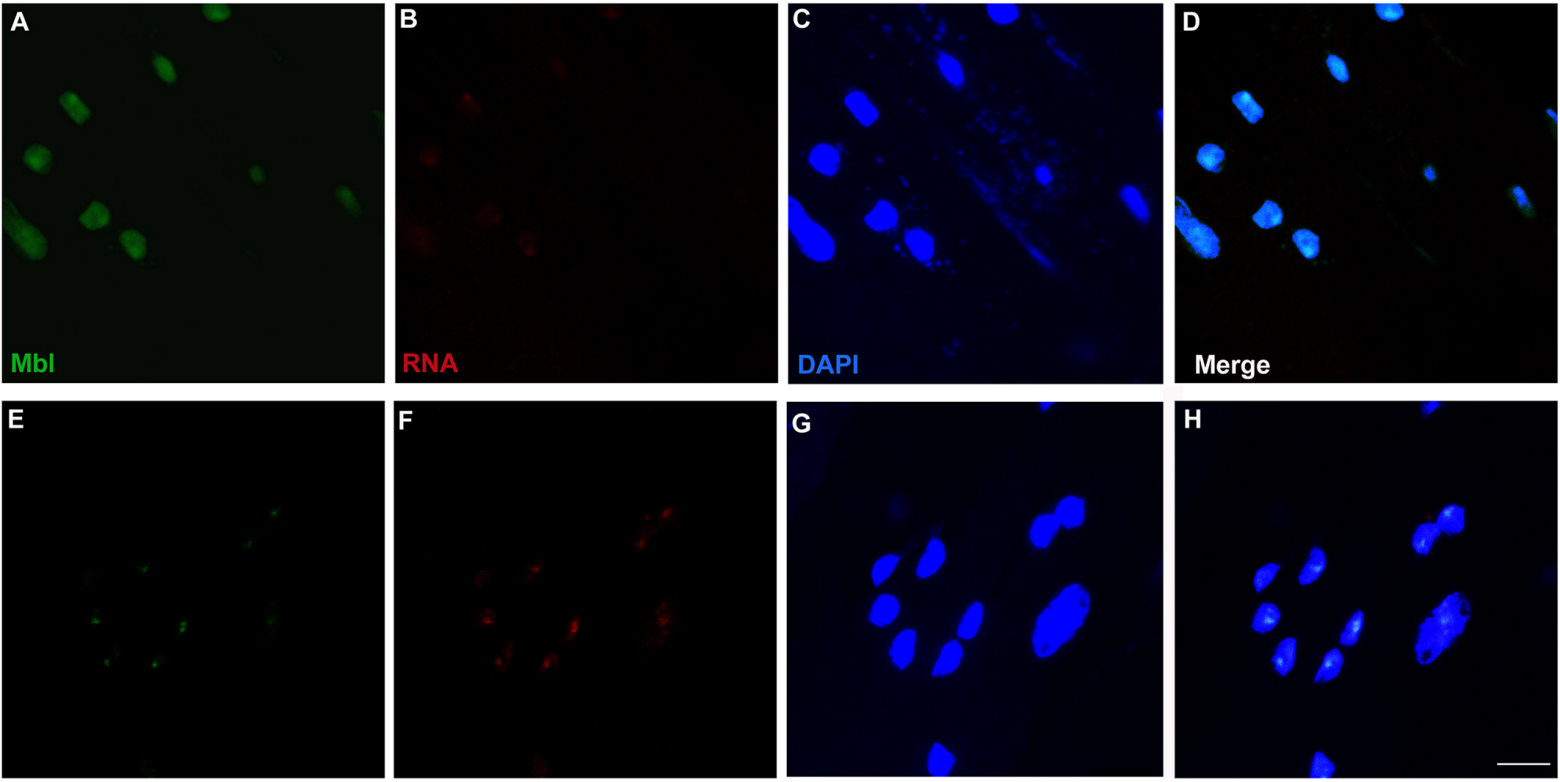
**Daunorubicin abrogates foci presence and redistributes Mbl in *Drosophila* cardiomyocytes.** Representative confocal images of cardiomyocytes of *Drosophila* DM1 flies fed with daunorubicin (A-D) or DMSO (E-H). Acquisition settings were the same for images taken within the same channel. Double *in situ* hybridization and immunodetection of CUG RNA (red) and Mbl (green) showed that Mbl was dispersed in the nuclei of cardiomyocytes of flies fed with daunorubicin. Nuclei were counterstained with DAPI (blue). Scale bar: 10 µm.

**Fig. 5.**
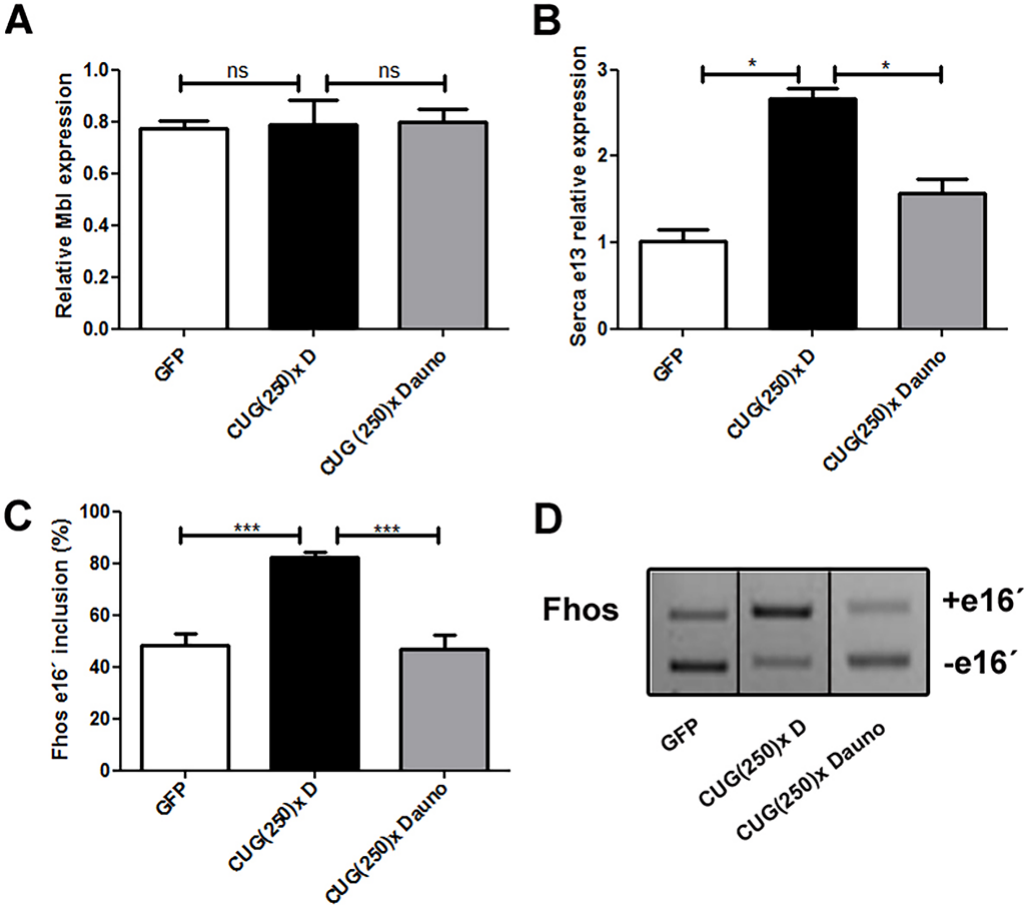
**In model flies fed with daunorubicin, Mbl transcript levels were not modified but Mbl-dependent splicing defects were restored.** (A) RT-qPCR to assess Mbl expression levels relative to endogenous tubulin in cardiomyocytes showed that it was not modified by administration of daunorubicin. (B) RT-qPCR results of *Serca* exon 13 expression relative to *Rp49* confirmed a significant rescue of the expression of this exon in model flies fed with daunorubicin. (C) Semi-quantitative PCR to assess inclusion of *Fhos* exon 16′ in model flies fed with DMSO or daunorubicin. *Rp49* transcripts were detected as endogenous control. Quantification of percentage of exon inclusion (see D for a representative result) confirmed an improvement of *Fhos* mis-splicing in model flies fed with daunorubicin. *P<0.05, ***P<0.001 (Student's *t*-test).

To observe the effect of daunorubicin on Mbl distribution, we performed FISH followed by immunofluorescence to detect CUG RNA foci and Mbl, respectively, in hearts of flies expressing expanded CUG repeats fed with 1 μM daunorubicin. As previously reported (Chakraborty et al., 2015), ribonuclear foci containing Mbl were present in the cardiac muscle nuclei of flies expressing CUG repeats. In cardiomyocytes of model flies fed with daunorubicin, we did not observe ribonuclear foci and Mbl was found homogeneously distributed in the nuclei (Fig. 4). As control, quantitative reverse transcription PCR (RT-qPCR) indicates that Mbl expression was not modified in DM1 flies treated with daunorubicin compared to DM1 flies fed or not with DMSO (Fig. 5A).

Next, we studied Mbl-dependent splicing events to test whether Mbl redistribution in the nuclei was enough to correct mis-splicing. Notably, daunorubicin corrects both *Serca* exon 13 (Fig. 5B) and *formin* (*Fhos*) exon 16′ (Fig. 5C,D) splicing events, which are Mbl-dependent events altered in heart of DM1 flies (Cerro-Herreros et al., 2017). These data show that daunorubicin induces Mbl redistribution in the nuclei enough to correct Mbl-dependent splicing events. In contrast, in flies expressing GFP in cardiomyocytes as controls, daunorubicin treatment did not alter the pattern of these splicing events, thus ruling out a general effect of daunorubicin on splicing regulation (Fig. S3B,C).

### Daunorubicin interacts directly with CUG RNA repeats, modifying its stability

**Fig. 6.**
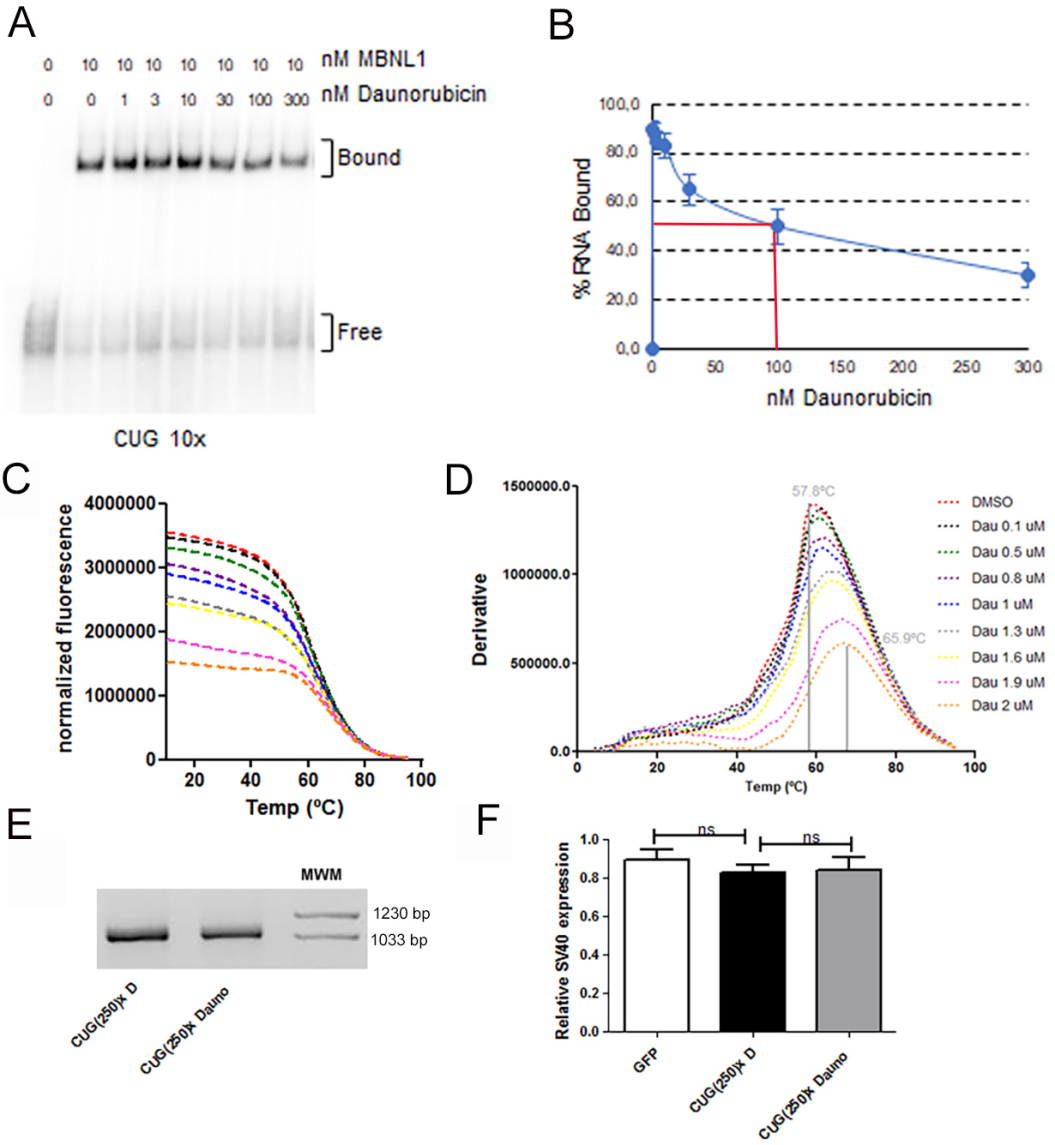
**Daunorubicin binds directly to CUG RNA, stabilizes CUG RNA structure *in vitro* and does not modify the CUG-repeat expression level or length.** (A) Gel-shift assays of 10 nM of purified bacterial recombinant GST-MBNL1 with 10 pM (2000 CPM) of uniformly [alphaP^32^] internally labelled *in vitro* transcribed RNAs containing 10 CUG repeats in the presence of increasing amounts of daunorubicin. (B) Gel-shift quantification with IC50 represented by the red lines. (C) Normalized fluorescence intensity and (D) first derivatives of RiboGreen fluorescence versus temperature at different daunorubicin concentrations. Grey vertical bars mark the minimum (57.8°C) and maximum (65.9°C) melting temperatures. (E) Agarose gel showing CTG repeat length inserted in the genomic DNA of DM1 flies fed with either DMSO or daunorubicin. MWM, molecular weight marker. (F) Bar graph represents means±s.e.m. of SV40 expression (contained in the CTG transgene) relative to tubulin. According to E and F, the effect of daunorubicin is not mediated by a reduction in the CTG repeat length or expression level in model flies. ns, not significant according to Student's *t*-test.

To test whether daunorubicin exerts its correcting effect by direct interaction with the CUG RNA, and competition with MBNL1, we performed electrophoretic mobility shift assay and analyzed the binding of recombinant GST-MBNL1 to internally labelled *in vitro* transcribed RNAs containing ten CUG repeats in the presence of increasing amounts of daunorubicin (Fig. 6A,B). According to these experiments, the quantity of daunorubicin needed to release half of MBNL bound to CUG 10× (*in vitro* IC50) would be 100 nM. We also used differential scanning fluorimetry (DSF) to monitor CUG-expanded RNA thermodynamics in the presence of growing concentrations of daunorubicin. The effect of a compound on RNA stability can be measured by DSF because RNA undergoes structural transitions from being double-stranded to single-stranded upon thermal unfolding. These transitions are accompanied by increased availability of binding sites for the single-stranded-RNA-specific dye RiboGreen. In this type of assay, the initial fluorescence originates from binding of the dye to unstructured, single-stranded, binding-competent regions of the RNA. After an initial decrease, the fluorescence increases because of destabilization of compact RNA structures, thereby becoming competent to bind the dye and achieving a point of maximal fluorescence. Then, this process reverses due to the formation of secondary structures indicated by a decrease in the binding affinity of the dye until RNA is completely folded (Silvers et al., 2015). We represented the fluorescence intensity and the first derivatives of normalized fluorescence of RiboGreen with an RNA probe containing 12 repeats of CUG versus temperature in the presence of concentrations of daunorubicin ranging from 0.1 to 2 µM and observed an important decrease in the maximal fluorescence and a shift of the melting towards higher temperatures with growing concentrations of daunorubicin (Fig. 6C,D). These data are coherent with the known intercalation of daunorubicin with double-stranded RNA (Doskocil and Fric, 1973) and suggest that daunorubicin stabilizes a double-stranded hairpin conformation of the CUG RNA repeats, resulting in less available single-stranded binding sites for the RiboGreen dye. Extrapolating these results to the binding of MBNL1 to expanded CUG repeats, it is likely that daunorubicin, by stabilizing a double-stranded hairpin RNA structure, limits the accessibility of MBNL1 to free single-stranded YGC RNA motifs (Lambert et al., 2014; Delorimier et al., 2014; Park et al., 2017).

Given the ability of daunorubicin to intercalate within the DNA and thus to alter transcription and replication, we also quantified the expression level and length of expanded CTG repeats. PCR amplification revealed that the DNA region including the CTG repeats had the same length (1178 bp) in DM1 flies fed with either DMSO or daunorubicin (Fig. 6E). Furthermore, quantitative RT-qPCR indicated no changes of CTG transgene expression in DM1 flies fed with DMSO compared to daunorubicin treatment (Fig. 6F). These data support a mechanism of action based on daunorubicin’s direct interaction with CUG RNA decreasing MBNL accessibility to the repeats, rather than a modification of the mutant DNA expression or length.

## DISCUSSION

We previously reported alterations of different heart functions, including heart rate, rhythmicity, SI, DI and %FS in flies expressing pure expanded CUG repeats (Chakraborty et al., 2015). The relevance of our model of heart dysfunction in DM1 was supported by the similarities between the cardiac phenotype in DM1 model flies and those documented in DM1 patients. Here, we show that the sole expression of Mbl can rescue all these cardiac parameters to their normal values, except the DI, which was not altered by MblC overexpression. These results support the involvement of Mbl in the cardiac dysfunction in DM1 and the therapeutic potential of its overexpression. Our data are consistent with the compound loss of Mbnl1 and Mbnl2 that evokes DM1-like cardiac phenotypes in mice (Lee et al., 2013). Furthermore, we identified pharmacological compounds reducing MBNL1 sequestration by expanded CUG repeats and found that one of these drugs, daunorubicin hydrochloride, achieved a complete rescue of cardiac parameters, including survival, arrhythmia, %FS and HP, with an increase of both DI and SI in DM1 flies. The fact that DI was suppressed by daunorubicin and not by MblC suggested that either other Mbl isoforms are required for a complete rescue of Mbl function in heart or that daunorubicin affects other Mbl-independent components, which might be involved in DI duration in DM1. Potential Mbl-independent activity of daunorubicin might be related to its ability to cause DNA damage. Daunorubicin belongs to the anthracyclines family, which has been used in curative and palliative treatment of several types of cancer (Hande, 1998; Gewirtz, 1999). Although anthracyclines produce a wide range of biological reactions, their primary mechanism of tumour cytotoxicity has been ascribed to the inhibition of the topoisomerase II enzyme (Nielsen et al., 1996; Arcamone et al., 1997). Thus, anthracycline drugs may cause DNA damage such as fragmentation and single-strand breaks, which can influence microsatellite expanded repeat instability. Notably, treatment of cells with 1, 2 and 5 mg/ml (1.8 μM, 3.54 μM and 8.86 μM, respectively) of doxorubicin, a 14-hydroxylated version of daunorubicin, causes some reduction of CTG repeat length (Hashem et al., 2004). However, we assessed CTG repeat length in 1 µM daunorubicin-treated flies and found no alterations. Of interest, the normal dosage used for daunorubicin hydrochloride in adults under 60 years of age is 45 mg/m^2^ once a day on days 1, 2 and 3 for the first course. This dosage would involve a concentration of 2 µM daunorubicin. DM1 flies fed with 1 µM daunorubicin throughout their lifespan have improved survival in comparison to DM1 flies fed with DMSO, suggesting that the cumulative daunorubicin doses achieved were not overtly toxic at this concentration.

Daunorubicin also interacts with double-stranded RNA (Doskocil and Fric, 1973). Our results indicate that daunorubicin directly interacts with expanded CUG RNA repeats and impairs binding of MBNL1 to these repeats, thus promoting MBNL1 release from CUG RNA foci in DM1 cells, as well as in DM1 *Drosophila* cardiomyocytes. Importantly, this reduced depletion of Mbl is enough to revert Mbl-dependent splicing events in DM1 flies. Overall, these results support a key role of Mbl protein sequestration in the cardiac dysfunctions observed in DM1.

Daunorubicin should be taken cautiously because of the genotoxic effects of this compound in long-term treatments. However, as proof of concept, our results suggest that inhibiting MBNL1 binding to expanded CUG repeats is a valid strategy to rescue cardiac phenotypes in DM1.

This study supports an important role for Mbl sequestration in the cardiac dysfunctions induced by expanded CUG repeats in DM. Furthermore, we uncovered a new FDA-approved compound with the ability of dissolving foci and competing MBNL1 away from CUG RNA foci. However, given the toxicity profile of daunorubicin, subsequent studies on murine models of the disease would be required to determine whether it could be considered as a suitable therapeutic approach for DM1.

## MATERIALS AND METHODS

### *Drosophila* strains

The cardiomyocyte-specific driver *GMH5-Gal4; UAS-GFP* was kindly provided by Dr Bodmer (Sanford Burham Institute, CA). Generation of CUG(250)× and UAS-MblC flies was previously described (Chakraborty et al., 2015; García-Casado et al., 2002). UAS-GFP strain was obtained from the Bloomington *Drosophila* Stock Center (Indiana University, Bloomington, IN).

### Compound treatment

Daunorubicin hydrochloride (Sigma) was added to the standard fly food to a final concentration of 1 µM in 0.1% DMSO. The control group was fed with 0.1% DMSO in standard food. Flies were transferred every 3 days to new fresh food media, with or without compound, for the duration of their whole lifespan in survival curve experiments or during 7 days in the case of the group used for cardiac analysis and immunofluorescence.

### Survival analyses

For survival analyses, a minimum of 50 flies were included per group and the survival curves were obtained at 29°C. Scoring of death was done as previously described (Chakraborty et al., 2015; Cerro-Herreros et al., 2017).

### Cardiac physiological analysis

For the physiological analysis, female flies were collected just after eclosion and were maintained for 7 days at 29°C in compound and DMSO-supplemented food. For the heart beat recordings, semi-intact heart preparations were made as previously described (Magny et al., 2013; Cerro-Herreros et al., 2017; Chakraborty et al., 2015). A Leica microscope, connected to an ORCA Flash (Hamamatsu) high-speed digital camera was used to take 20 s recordings at a minimum speed of 150 frames/s. Different cardiac parameters were measured using SOHA software (Ocorr et al., 2014).

### Alternative splicing quantification

For each biological replicate, total RNA was extracted using Qiazol (Qiagen) from at least 20 female hearts. One microgram of RNA was digested with DNase I (Invitrogen) and retro-transcribed with SuperScript II (Invitrogen) using random hexanucleotides. To analyze *Fhos* exon 16′ inclusion, 20 ng of cDNA was used in a standard semi-quantitative PCR reaction with GoTaq polymerase. *Serca* exon 13 expression, SV40 to measure repeats expression, and Mbl expression were checked with RT-qPCR as previously described (Cerro-Herreros et al., 2017, 2016).

### Repeat number detection

To confirm the number of repeats in the flies fed with the compound or DMSO, 40 ng of genomic DNA was used as a template for the PCR amplification as described previously (Chakraborty et al., 2015). The region amplified by the primers includes long repeats (750 bp) and 428 bp of the CTG (250)× plasmid. PCR amplification was performed under the following conditions: 95°C for 2 min, followed by 30 cycles of 98°C for 20 s, 65°C for 30 s, 72°C for 1 min and final extension at 72°C for 5 min. The PCR products were analyzed by electrophoresis at 110 V in 1.5% agarose gels.

### DM1 myoblast culture and staining

Primary human myoblast cells originating from muscle biopsies of genetically confirmed DM1 patients were maintained at 37°C with 10% CO_2_ in skeletal muscle cell basal media with supplements (PromoCell, Heidelberg, Germany) and 10% fetal calf serum. For myoblast differentiation, cells were maintained for 4 days in DMEM with 2% fetal calf serum, the drug was added for 16 h and then assayed for CUG RNA foci and MBNL1 localization by classic RNA FISH immunofluorescence. Glass coverslips containing plated cells were fixed in PFA 4% for 15 min and washed two times with PBS. The coverslips or slides were incubated for 10 min in PBS plus 0.5% Triton X-100 and washed three times with PBS before pre-hybridization in 40% DMSO, 40% formamide, 10% BSA (10 mg/ml), 2× SCC for 30 min. The coverslips or slides were hybridized for 2 h in 40% formamide, 10% DMSO, 2× SCC, 2 mM vanadyl ribonucleoside, 60 μg/ml tRNA, 30 μg/ml BSA plus 0.75 μg CAG8x-Cy3 DNA oligonucleotide probe (Sigma). Following FISH, the coverslips or slide were washed twice successively in 2× SCC/50% formamide, in 2× SCC and in PBS. The coverslips or slides were incubated for 2 h with primary polyclonal antibody against MBNL1 (1/100 dilution, gift of Prof. Charles Thornton, University of Rochester Medical Center, Rochester, NY). Slides or coverslips were washed twice with PBS before incubation with a goat anti-rabbit secondary antibody conjugated with Alexa Fluor 488 (1/500 dilution, #A32731, Fisher Scientific SA) for 60 min, incubated for 10 min in 2× SCC/DAPI (1/10,000 dilution) and rinsed twice in 2× SSC before mounting in Pro-Long media (Molecular Probes). Slides were examined using a fluorescence microscope (Leica).

### Foci quantification in DM1 fibroblasts

Immortalized (hTERT) human DM1 (1300 CTG repeats) skin fibroblasts conditionally expressing MyoD were provided by D. Furling's laboratory in the Institute of Myologie, Paris. Fibroblast cells were grown in Dulbecco's modified Eagle medium (DMEM) with 4.5 g/l glucose, 1% penicillin and streptomycin (P/S), and 10% fetal bovine serum (FBS; Sigma). Fibroblasts were aliquoted into 96-well plates (1.0×104 cells per well), incubated with daunorubicin or DMSO (48 h) and fixed in 4% PFA for 10 min at room temperature followed by several washes in 1× PBS. Fixed cells were incubated in pre-hybridization buffer (2× SSC, 30% deionized formamide) for 10 min at room temperature and hybridized with Cy3-(CAG)_7_-Cy3-labelled probe diluted 1:500 in hybridization buffer [40% formamide, 2× SSC, 0.2% BSA, 10% dextran sulfate, 2 mM ribonucleoside-vanadyl complex, 10% tRNA (10 mg/ml) and 10% herring sperm] for 2 h at 37°C. After hybridization, cells were washed twice with pre-hybridization buffer for 15 min at 45°C, washed twice with 0.5× SSC for 5 min at 37°C, washed with 1× PBS for 15 min at room temperature, incubated with Hoechst 33342 (5 mg/ml) diluted 1:2000 in 1× PBS for 20 min at room temperature, and mounted with 20% Mowiol. Images were taken and analyzed using an IN Cell Analyzer 2200 Imaging System.

### Immunofluorescence analysis in flies

For immunofluorescence analysis in *Drosophila* cardiomyocytes, 7-day-old dissected female fly hearts were fixed for 20 min in 4% paraformaldehyde and washed in PBT (PBS containing 0.3% Triton X-100) before staining. Mbl staining and FISH to detect ribonuclear CUG foci were performed as previously described (Llamusi et al., 2012). All the confocal images were taken with an Olympus FV1000 microscope.

### Differential scanning fluorimetry (DSF)

DSF experiments were performed to understand the interaction of daunorubicin with the double-stranded CUG hairpin using DSF technique (Silvers et al., 2015). The experiment was performed using a StepOnePlus Real-Time PCR system (Life Technologies) with the melting curve software to measure the fluorescence intensity. A MicroAmp^®^ Fast Optical 96-well plate (Life Technologies) was used with 50 μl of solution per well. The RiboGreen dye was used at a final concentration of 300 nM, whereas the synthetic double-stranded CUG RNA (12× CUG) was used at a final concentration of 600 nM. For each compound (in this case daunorubicin and DMSO), four technical replicates were performed and, for daunorubicin, eight different concentrations ranging from 0.1-2 μM were used. Sodium cacodylate buffer was used at pH 6.1, which is essential for experiments using RNA. During the DSF experiment, the temperature was increased from 4 to 95°C at an increment of 0.2°C with an equilibration time of 5 s at each temperature prior to measurement. The Excel sheets containing melting temperature, and normalized and derivative fluorescence data were exported into GraphPad Prism 5 software for further analysis and generation of graphics.

### Electrophoretic mobility shift assay

10 pM (2000 CPM) of labelled CUG 10× RNA was incubated at 90°C for 5 min in binding buffer [BB; 0.75 mM MgCl_2_, 50 mM Tris-HCl (pH 7.0), 75 mM NaCl, 37.5 mM KCl, 5.25 mM DTT, 0.1 mg/ml BSA, 0.1 mg/ml Bulk tRNA] and allowed to cool to room temperature. After cooling, RNasin was added to a final concentration of 0.4 U/μl. GST-MBNL1^Δ101^ was then added and the mixture was incubated on ice for 20 min. The solution mixture was loaded onto a non-denaturing 6.0% (w/v) polyacrylamide gel (acrylamide/bisacrylamide, 40:1, w/w) containing 0.5× TBE [1× TBE is 90 mM Tris-base, 89 mM boric acid and 2 mM EDTA (pH 8.0)], which had been pre-electrophoresed at 110 V for 20 min at 4°C. The gel was electrophoresed at 110 V at 4°C for 3 h, then dried and exposed to a phosphorimager screen and imaged using a Typhoon 9410.

## Supplementary Material

Supplementary information
