# Correction: Daunorubicin reduces MBNL1 sequestration caused by CUG-repeat expansion and rescues cardiac dysfunctions in a *Drosophila* model of myotonic dystrophy (doi: 10.1242/dmm.032557)

**DOI:** 10.1242/dmm.035501

**Published:** 2018-05-18

Mouli Chakraborty, Chantal Sellier, Michel Ney, Pascal Villa, Nicolas Charlet-Berguerand, Ruben Artero, Beatriz Llamusi

There was an error published in Dis. Model Mech. 11, dmm032557 (doi: 10.1242/dmm.032557)

The name of the co-author Pascal Villa was presented incorrectly. It has now been corrected and the original article changed correspondingly.

