# Supplementary material for: Daunorubicin reduces MBNL1 sequestration caused by CUG-repeat expansion and rescues cardiac dysfunctions in a Drosophila model of myotonic dystrophy
Source: Dis Model Mech. 2018 Apr 23;11(4):dmm032557. doi: 10.1242/dmm.032557 (PMC5963859; doi:10.1242/dmm.032557)
Supplement: Supplementary information [file dmm-11-032557-s1.pdf]

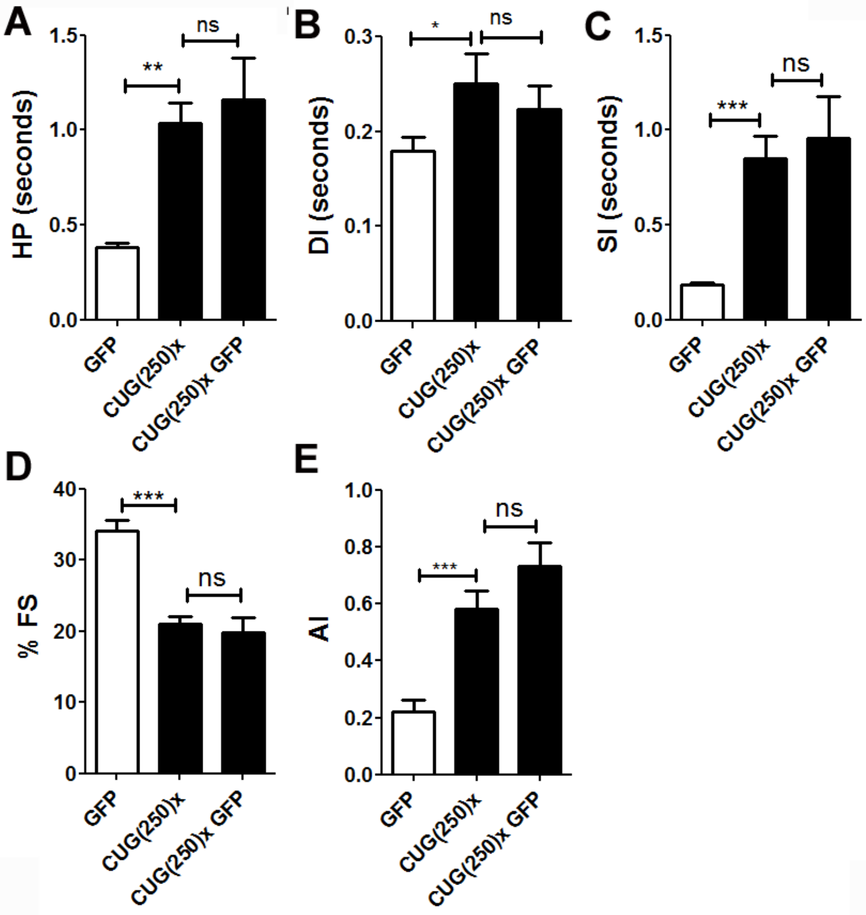


**Supplemental figure S1: GFP expression in cardiomyocytes did not alter cardiac performance.** HP, SI, DI, FS, and AI of model flies expressing or not GFP in cardiomyocytes were not significantly different. (n used was between 24 and 32). ***$p<0.001$, **$p<0.01$, *$p<0.05$, ns= not significant (Student´s t-test)

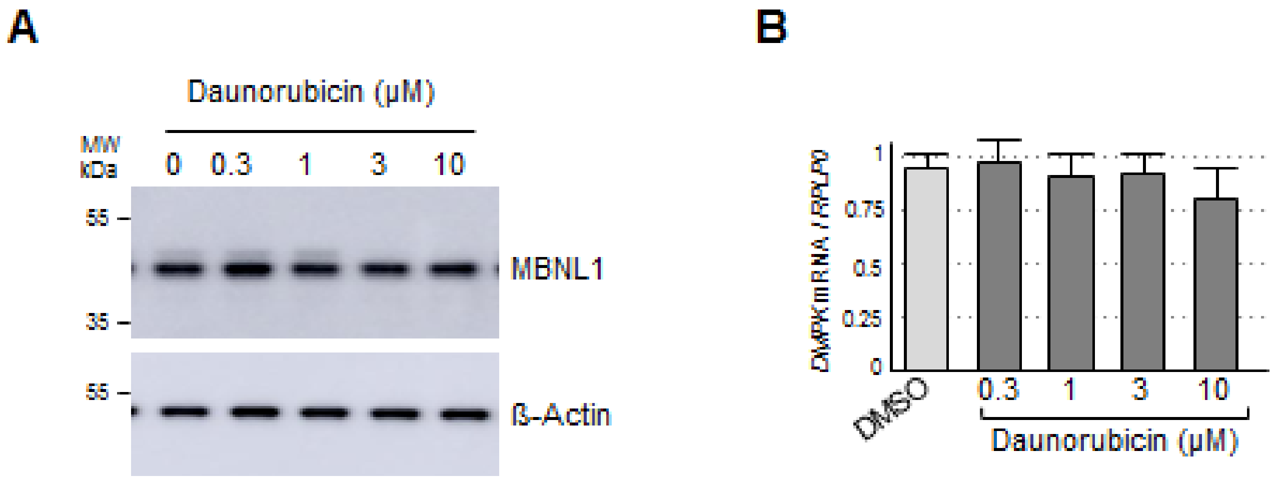


**Supplemental figure S2: Daunorubicin did not modify MBNL1 or DMPK expression levels in human DM1 myoblasts.** Immunoblotting against MBNL1 and ß-Actin of DM1 myoblasts cell lysate upon Daunorubicin treatment at 0.3, 1, 3 and 10 µM in DMSO for 24 h. (B) Quantitative RT-qPCR of DMPK and RPLP0 mRNAs of DM1 myoblasts total RNA upon Daunorubicin treatment at 0.3, 1, 3 and 10 µM in DMSO for 24 hours.

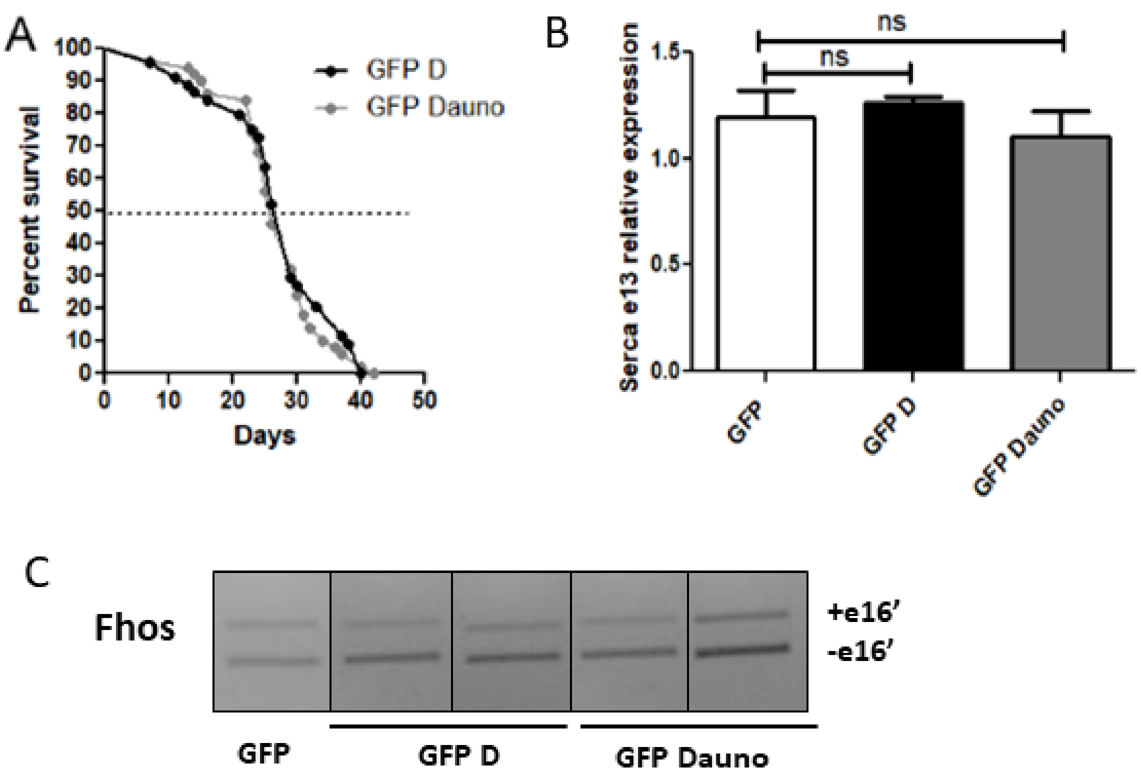


**Supplemental figure S3: Daunorubicin did neither modify survival nor Mbl-dependent splicing in control flies expressing GFP.** (A) Survival curves of control flies fed with DMSO (D) or Daunorubicin (Dauno) were identical. (B) qRT-PCR results of *Serca* exon 13 expression relative to *Rp49* confirmed that Daunorubicin did not influence this splicing pattern. The histograms show the mean±SEM. ns- not significant. (C) Semiquantitative RT-PCR to assess inclusion of *Fhos* exon 16′ in control flies fed with DMSO or Daunorubicin. *p < 0.05, **p < 0.01, ***p < 0.001 (Student's t-test).
